# The role of spatial boundaries in shaping long-term event representations

**DOI:** 10.1016/j.cognition.2016.05.013

**Published:** 2016-09

**Authors:** Aidan J. Horner, James A. Bisby, Aijing Wang, Katrina Bogus, Neil Burgess

**Affiliations:** a UCL Institute of Cognitive Neuroscience, 17 Queen Sq., London WC1N 3AR, UK; b UCL Institute of Neurology, Queen Sq., London WC1 3BG, UK; c Department of Psychology, University of York, York YO10 5DD, UK

**Keywords:** Episodic memory, Event segmentation, Spatial memory, Computational modelling, Virtual reality

## Abstract

When remembering the past, we typically recall ‘events’ that are bounded in time and space. However, as we navigate our environment our senses receive a continuous stream of information. How do we create discrete long-term episodic memories from continuous input? Although previous research has provided evidence for a role of spatial boundaries in the online segmentation of our sensory experience within working memory, it is not known how this segmentation contributes to subsequent long-term episodic memory. Here we show that the presence of a spatial boundary at encoding (a doorway between two rooms) impairs participants’ later ability to remember the order that objects were presented in. A sequence of two objects presented in the same room in a virtual reality environment is more accurately remembered than a sequence of two objects presented in adjoining rooms. The results are captured by a simple model in which items are associated to a context representation that changes gradually over time, and changes more rapidly when crossing a spatial boundary. We therefore provide the first evidence that the structure of long-term episodic memory is shaped by the presence of a spatial boundary and provide constraints on the nature of the interaction between working memory and long-term memory.

## 1 Introduction

As we move through our environment our senses receive a continuous stream of information, yet our memory of the past is subjectively discrete in nature. We typically recall single instances in time and space. These discrete ‘event engrams’ are thought to be the fundamental unit of episodic memory (Tulving, 1983), allowing us to re-experience or recollect previous life events (Aggleton and Brown, 1999, Jacoby, 1991, Yonelinas, 1994). Thus, our continuous sensory experience must be segmented in order to encode these more discrete episodic events. Previous research has shown that spatial boundaries play a key role in the online segmentation of events, and this segmentation affects short-term memory (Radvansky and Copeland, 2006, Radvansky et al., 2010). However, it is not known how spatial boundaries affect the structure of long-term episodic memory.

When asked to watch a short video clip, participants are readily able to segment the video into ‘events’, with close agreement across participants on the location of these event boundaries (Newtson, 1973, Newtson and Engquist, 1976). This event segmentation process is thought to be automatic in nature, with consistent neural responses in a network of cortical regions in the presence of an event boundary, despite participants passively watching videos (Zacks et al., 2001). The ‘event segmentation theory’ proposes that these boundaries are defined by an increase in temporal prediction error (Reynolds et al., 2007, Zacks et al., 2007). Participants perceive an event boundary when they are unable to predict what is about to happen (Zacks, Kurby, Eisenberg, & Haroutunian, 2011).

However, these studies are agnostic in relation to what drives this prediction error and therefore the perception of an event boundary. Put simply, what environmental factors contribute to the presence of an event boundary? Research related to the reading of narratives suggests that readers automatically create representations analogous to those that would be created in real life. These ‘situation models’ are thought to aid the reader in understanding and interpreting the narrative of written text (van Dijk and Kintsch, 1983, Zwaan, 1999, Zwaan and Radvansky, 1998). Within this literature, the ‘event-indexing model’ suggests that these situation models are centred on ‘events’ (Zwaan, Langston, & Graesser, 1995). Importantly, they suggest that events are segmented based on five specific dimensions, most notably both space and time (also see Rinck and Bower, 2000, Rinck et al., 1997). This emphasis on both space and time accords with the idea that human episodic memory is inherently spatiotemporal in nature (O’Keefe and Nadel, 1978, Tulving, 1983). Therefore, whether watching a video or reading a narrative, we segment our sensory experience into ‘events’ based on the spatiotemporal characteristics of the environment. Here we concentrate on the role of spatial boundaries in event segmentation, and its effect on long-term episodic memory.

The presence of event boundaries has been shown to affect short-term memory. When watching videos depicting a series of events with embedded objects, participants are better at remembering objects if they are still watching the same event relative to if they are watching a subsequent event (Swallow, Zacks, & Abrams, 2009). Similarly, when reading narratives, participants are better at remembering words they have seen just before reading the phrase “a while later” relative to “an hour later” (Speer & Zacks, 2005). The implied passage of time in “an hour later” impaired participants’ performance for words they had just read.

Short-term memory has also been shown to be disrupted in the presence of a spatial boundary – in this case, a doorway. When participants navigate a series of rooms in a virtual reality (VR) environment, picking up and placing down objects in each room, memory for the object they are carrying is more accurate when tested in the same room relative to when tested in an adjoining room (the ‘location updating effect’; Radvansky & Copeland, 2006). This effect is not simply due to being tested in the same relative to a different context (in this case, a room) (i.e., a “context-dependent” memory effect; Gooden and Baddeley, 1975, Thomson and Tulving, 1970), as performance was also impaired if participants returned to the original room after moving to a new room (Radvansky, Krawietz, & Tamplin, 2011). Importantly, as the same effect is seen when participants physically walk (Radvansky et al., 2011) or imagine walking (Lawrence & Peterson, 2014) from one room to another, the effect is not specific to VR environments. Although the presence of a spatial boundary can affect short-term memory, it is not clear whether longer lasting effects are seen in relation to episodic memory (see Section 6 for how the short-term ‘location updating effect’ could affect long-term memory). In sum, the presence of an event boundary, in narratives, videos and VR environments, can disrupt short-term memory, suggesting a link between event segmentation and subsequent memory.

There is also a close correspondence between event segmentation and long-term memory. The ability to consistently segment videos into events is correlated with participants ability to remember those events later in time (Sargent et al., 2013). Further, participants memory for video narratives is disrupted when scenes including event boundaries are removed (Schwan & Garsoffky, 2004). Thus, remembering entire narratives is aided by the presence of event boundaries.

The key question we wish to address here is how the presence of an event boundary affects long-term memory within a specific event. For example, if participants encounter two objects that are separated in both time and space, is the association between these objects modulated by whether or not they were encountered in the same event? Ezzyat and Davachi (2011) addressed this question, manipulating the suggested time between two actions with the words “a moment later” vs “a while later”. When later cued with an action, participants were less accurate at remembering the next action in the narrative when they were separated by the words “a while later” relative to “a moment later”. Therefore, it is harder to remember two sequential actions separated by an event boundary than two actions that occurred in the same event. More recently, the presence of an event boundary has been shown to affect memory for the temporal order of objects (DuBrow & Davachi, 2013). Participants were presented with a series of faces and objects and performed a different categorisation task for each stimulus type. Event boundaries were defined as a switch in both stimulus type and categorisation task. When later tested with a recency discrimination judgement (“which of these two stimuli were seen first?”), performance was impaired if an event boundary had been encountered between the two stimuli at encoding. These results are consistent with the idea that stimuli encountered within an event are more tightly associated than those encountered across events.

Here we wanted to assess the role of spatial boundaries in relation to long-term episodic memory. By focussing on how space shapes long-term memory we are able to draw upon existing knowledge of the underlying neural architecture of the medial temporal lobes (MTL), including the spatially modulated firing characteristics of specific neurons (Hafting et al., 2005, Lever et al., 2009, O’Keefe and Dostrovsky, 1971, Solstad et al., 2008). Of particular interest, neurons in the MTL fire in relation to the rodents proximity to a spatial boundary (Lever et al., 2009, O’Keefe and Burgess, 1996, Solstad et al., 2008) and place cells may cluster around doorways in multi-compartment environments (Spiers, Hayman, Jovalekic, Marozzi, & Jeffery, 2013). This preferential coding of boundary information might, in part, be driven by the behavioural relevance of boundaries, allowing for the MTL system to appropriately compartmentalise space (Stachenfeld, Botvinick, & Gershman, 2014). Finally, we can start to link between experimental evidence for ‘contextual’ signals in the hippocampus, and how they change across time (Mankin et al., 2015, Manns et al., 2007) with psychological models of temporal memory (e.g., Burgess and Hitch, 2005, Estes, 1950, Howard and Kahana, 2002), to further understand how the MTL supports long-term episodic memory.

We adopted the approach introduced by Radvansky and colleagues (e.g., Radvansky & Copeland, 2006), requiring participants to navigate through a series of rooms in a VR environment. However, similar to DuBrow and Davachi (2013), we were interested in long-term memory for the temporal order of objects. Therefore, we presented two objects in each room, separated in both time and space. Following encoding, participants performed several memory tasks (see Methods). Critically, we assessed temporal memory by presenting a single object and asking “which object came next?” or “which object came immediately before?”. For half of these trials, the cued and retrieved objects were encountered in the same room, whereas for the other half they were encountered in adjoining rooms (i.e., separated by a spatial boundary). In Experiment 1, we found evidence that temporal memory for two sequentially presented objects was more accurate when the objects were encountered in the same room relative to adjoining rooms. Experiment 2 replicated this effect, controlling for the distance between each object and the time between interacting with each object. Experiment 3 replicated the effect, whilst further controlling for the time between first seeing each object. We therefore provide the first evidence that spatial boundaries play a key role in shaping the content of long-term episodic event representations.

## 2 Experiment 1

### 2.1 Methods

#### 2.1.1 Participants

43 participants (25 male) were recruited through the online UCL Psychology Subject Pool. 22 were assigned to the “which object came next?” group (Group 1) and 21 to the “which object came before?” group (Group 2). By self-report 4 participants were left-handed and the remainder right-handed. All participants gave informed consent and were reimbursed for their time (£7.50). The experiment was approved by the UCL Institute of Cognitive Neuroscience Departmental Research Ethics Committee (ICH-AH-PWB-2-10-13a).

In total, 3 participants did not finish the experiment, leaving 20 participants per group. Group 1 had a mean age of 23.2 (SD = 3.5), Group 2 had a mean age of 22.9 (SD = 3.3).

#### 2.1.2 Materials

The experiment used a desktop PC, with the VR environment displayed on a standard TFT monitor. For the VR study task, the rooms environment was created using Google SketchUp (http://www.sketchup.com/) and imported into Unity (https://unity3d.com/). There were 48 equally sized rooms, distinguishable by their wall-paper and floor colour. Each room contained two different tables (one per object, see below). Each table was placed relatively close to one of the doors to maximise the distance between tables within-room and minimise the distance between tables across-room. The 48 rooms were connected to each other via doorways with closed but navigable doors (i.e., it was possible to walk through the door into the next room). Each room was connected to two other rooms via separate doorways creating a single loop of 48 rooms. All scripting related to the encoding task was based in Unity.

144 colour objects were used, a subset of those used in Horner and Henson (2008), half man-made, half natural. 96 of these objects were shown in the VR Study task (two per room; 48 man-made, 48 natural). The remaining 48 objects were used as “new” items in the recognition task at Test (24 man-made, 24 natural). Object presentation at Study was fixed, so that all participants saw the same sequence of objects and rooms. Stimulus presentation at Test was controlled by the Cogent toolbox (http://www.vislab.ucl.ac.uk/cogent.php) in MATLAB. Presentation order at Test was randomly chosen for each participant.

#### 2.1.3 Procedure

##### 2.1.3.1 Study phase

Prior to Study, participants practiced the VR task in a separate 4-room environment with objects not used in the main experiment. They then practiced the test tasks with these same objects.

**Fig. 1:**
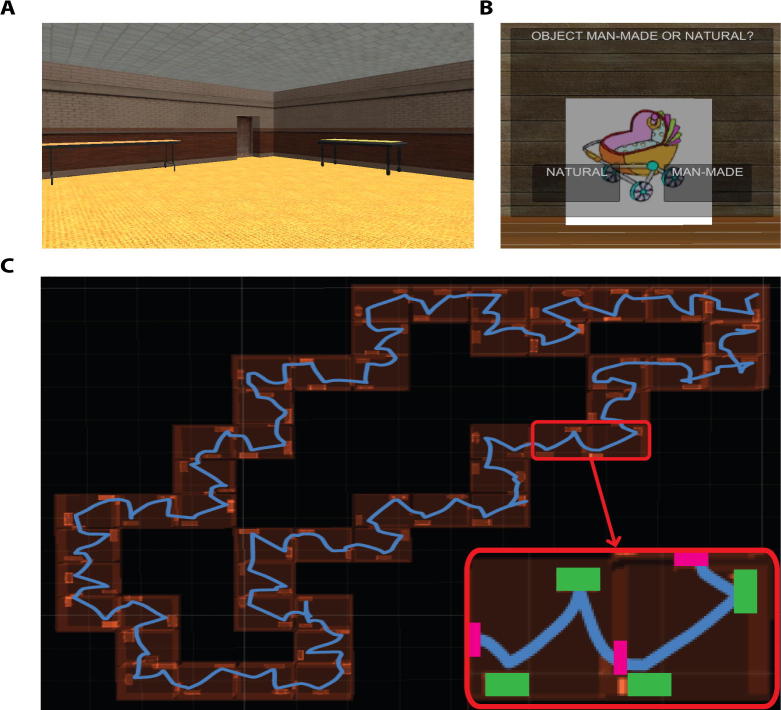
Virtual reality environment. Screenshots of (A) an example room in Experiment 1, showing a doorway and the two tables where objects were shown and (B) an example man-made/natural question when the participant approached an object. (C) An overhead view of the room layout in Experiment 1 including the locations of the tables, with an example path (in blue) taken by a participant through all 48 rooms. The bottom right hand corner zooms in on two rooms, showing the path (in blue), tables (in green) and doorways (in magenta). (For interpretation of the references to colour in this figure legend, the reader is referred to the web version of this article.)

At Study, participants were required to navigate through a VR environment (displayed on a computer screen in front of the participant from a first-person perspective) and were shown a series of objects embedded in this environment. Specifically, they navigated through a series of rooms, each separated by a closed door (Fig. 1). Objects were positioned in the centre of the tables within the rooms, with only one object present on one table at any one time. Participants were required to navigate to the object, using the arrow keys on a standard keyboard. Once positioned in front of the object, close to the edge of the table, a prompt box appeared in the centre of the screen with two response options: “man-made” or “natural”. Participants were required to press the “M” key if the object was man-made and the “N” key if the object was natural (mean accuracy = 98%). The object and response options were removed following the participant’s response, and the next object appeared on the next table in the sequence. As such, no two objects were seen at the same time. If the next object was located in the same room, participants were required to navigate to the location of the next object (i.e., the other table) without leaving the room. If the next object was located in the following room, participants had to walk through a doorway into the next room, before approaching the object. Participants moved through the rooms sequentially, without returning to any previous rooms. The order of objects at Study was fixed across participants.

##### 2.1.3.2 Test phase

**Fig. 2:**
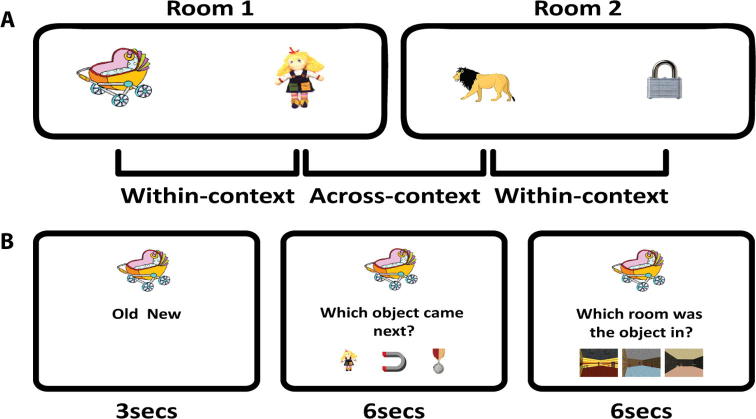
Experimental design. (A) An example object sequence at Study showing within-context and across-context object pairs. (B) Trail sequence at Test for an ‘old’ object, showing trial timings for the recognition, temporal order and context memory judgements. Timings shown are the maximum time during which participants were required to respond. Each trial began with a 500 ms fixation cross. Between each memory judgement the object stayed on the screen with no other text or stimuli for 500 ms. A blank screen was presented for 1500 ms at the end of each trial.

At Test, memory for an object was tested in three separate ways in a single trial: (1) recognition memory, (2) temporal memory and (3) context memory (Fig. 2). Object order at Test was randomised. Following a 500 ms fixation cross, a single object appeared in the middle of the screen on a grey background with the words “old” and “new” presented below the object to the left and right of centre respectively. The object was either “old” (seen at Study) or “new” (not previously seen in the experiment). There were 88 old objects (taken from rooms 3–46, see below) and 48 new objects. Participants were required to respond using buttons 1 (old) and 3 (new) on the keypad, within 3000 ms. If the object was new (irrespective of how the participant responded) a blank grey screen was presented for 1500 ms prior to the start of a new trial.

If the object was old (again, irrespective of how the participant responded) the object remained on screen and below this the question “which object came next?” (Group 1) or “which object came immediately before” (Group 2) appeared. Below the question three further old objects were presented to the left, middle and right of screen centre. One of the objects was the next (Group 1) or previous (Group 2) object in the encoding sequence. Participants had to select this object, ignoring the two ‘foils’. The location (left, middle or right) of the correct object on the screen was randomly chosen on each trial. One of the foils was randomly selected from any of the previous objects (i.e., seen before the cue object at Study), with the constraint that it was not seen in the same room as the cue, or an adjoining room. The other foil was randomly selected from any of the subsequent objects (i.e., seen after the cue object at Study), with the same constraint that it was not seen in the same rooms as the cue, or an adjoining room. This constraint was imposed so that none of the foils were taken from the same room as the cue or target object. This constraint meant we only tested memory for objects in rooms 3–46, hence there were only 88 old objects (rather than the full set of 96 seen at Study). Note, the selection of foils was not constrained by whether the object had been previously presented as a cue for the recognition judgement, thus it is possible that recognition performance may be inflated for certain objects if they were selected as a foil in the temporal memory task prior to being selected as a cue in the recognition task. However, this should apply equally to objects seen first or second in a room, so should not affect the relative difference between conditions.

For half the objects, the correct (to be selected) object was from the same room (within-context) and for the other half the correct object was from an adjoining room (across-context). For Group 1 (“which object came next?”), the first object in the room was always in the within-context condition (as the next object was the second object in the room) whereas the second object was always in the across-context condition (as the next object was the first object in the next room). This relationship between object number within a room and within- vs across-context temporal order judgement was reversed for Group 2 (“which object came before?”). Participants were required to respond using buttons 1–3 on the keypad, within 6000 ms.

Following the temporal memory judgement, the centrally presented old object remained on screen for 500 ms. The question “which room was the object in?” then appeared below the object. Three images of rooms from the Study task were then presented underneath this question. One of the images was of the room in which the object was originally presented. The location (left, middle or right) of the correct room was randomly chosen on each trial. One foil was from a preceding (but not adjacent) room and the other foil was from a subsequent (but not adjacent) room. Participants had to select the image of the room in which the object was originally seen using buttons 1–3 on the keypad, within 6000 ms. Following the context task, a grey screen was presented for 1500 ms prior to the start of a new trial. Participants were encouraged to respond on each trial, such that guessing was encouraged. If participants failed to respond to any of the tasks in the time given, that specific task component of the trial was marked as incorrect. Across participants, the mean percentage of trials where no response was given was low (3.3% for the recognition judgement, 0.99% for the temporal memory judgement and 0.63% for the context memory judgement).

#### 2.1.4 Statistical analyses

First, for temporal order memory, we report a 2 × 2 mixed ANOVA with the within-subject factor “Context” (within- vs across-context) and the between-subject factor “Direction”, relating to the direction of the temporal order question (i.e., which object came next/before). The first object in each room was assigned to the within-context condition in Group 1 and the across-context condition of Group 2 (and vice versa for the second object in each room). This analysis was designed to look for an effect of context, and to assess whether this effect varied as a function of the direction of the temporal order question being asked.

For the main recognition and context memory results, we report 2 × 2 mixed ANOVAs with the within-subject factor “Object”, relating to whether the object was seen first or second in the room, and the between-subject factor “Direction”, relating to the direction of the temporal order question (i.e., which object came next/before). These analyses were conducted to rule out any difference in either recognition or context memory in relation to whether objects were seen first or second within a room, and further whether this effect differed between the two participant groups. Note, the groups only differed in relation to the direction of the temporal order question, so no differences were predicted in relation to recognition or context memory. For completeness, we also present this analysis on temporal order memory (an interaction between Object and Direction in this analysis is identical to a main effect of Context in the main temporal order analysis described above).

For the main effects and interactions within each ANOVA we report partial-eta squared effect sizes (*η_p_*^2^). Significant interactions are further interrogated with paired *t*-tests. For these *t*-tests, we report Cohen’s *d* as the mean difference between conditions divided by the mean standard deviation across conditions (*d_av_*) (Cumming, 2012, Lakens, 2013). Given the specific predictions relating to temporal order memory, we report these data prior to the recognition and context memory data.

All data required for these analyses are freely available on Figshare (http://dx.doi.org/10.6084/m9.figshare.1609803.v3). The means for each participant across all conditions for the recognition memory, temporal order memory and context memory tasks, as well as the time and path distance across conditions, are included.

### 2.2 Results

#### 2.2.1 Temporal memory

**Fig. 3:**
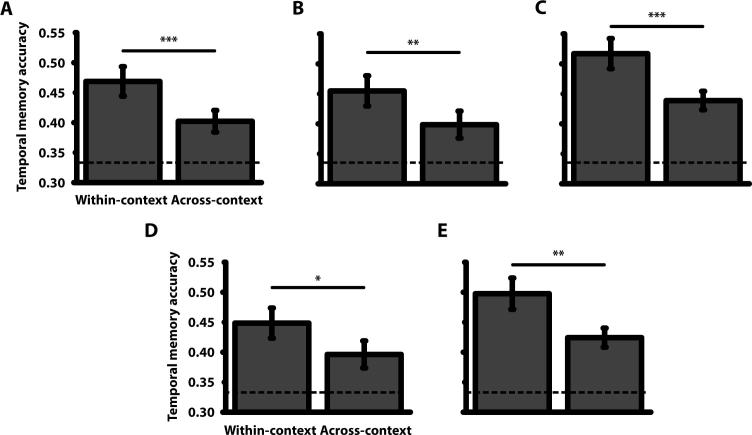
Temporal memory. Mean temporal accuracy for within-context and across-context object pairs (collapsed across the direction of the temporal question) for (A) Experiment 1, (B) Experiment 2 and (C) Experiment 3 as well as the control analyses for (D) Experiment 2 and (E) Experiment 3. Error bars represent ±1 standard error of the mean. The dotted line shows chance level of performance (0.33, given three-alternative forced choice). ^***^ p < 0.001, ^**^ p < 0.01, ^*^ p < 0.05.

Mean temporal accuracy was above chance at 44% (Table 1), *t*(39) = 5.46, p < 0.001, *d* = 0.86 (chance = 33%). A 2 × 2 mixed ANOVA with a within-subject factor of Context (within-context vs across-context) and a between-subject factor of Direction (“which object came next” – Group 1 vs “which object came before” – Group 2) revealed a main effect of Context, *F*(1, 38) = 12.50, p < 0.001, *η_p_*^2^ = 0.25, with higher accuracy in the within-context than across-context condition (Fig. 3A). This effect did not interact with Direction, *F*(1, 38) = 2.57, p = 0.12, *η_p_*^2^ = 0.06 (nor was there a main effect of Direction, *F*(1, 38) = 3.60, p = 0.07, *η_p_*^2^ = 0.09, though we note a trend for higher accuracy in the “which came next” (0.47) versus “which came before” (0.40) question). Participants were better at remembering the sequence of objects when both objects were seen in the same room relative to when they were seen in adjoining rooms. Crossing a spatial boundary, by walking through a doorway, therefore affects long-term memory for sequentially presented objects.

Finally, a 2 × 2 (Object × Direction) mixed ANOVA revealed a significant interaction, *F*(1, 38) = 12.50, p < 0.001, *η_p_*^2^ = 0.25 (identical to the main effect of Context seen above). No significant main effects of either Object, *F*(1, 38) = 2.57, p = 0.12, *η_p_*^2^ = 0.06, or Direction, *F*(1, 38) = 3.60, p = 0.07, *η_p_*^2^ = 0.09, were present.

#### 2.2.2 Recognition & context memory

Recognition accuracy was high, with a mean hit rate of 76% (though note the mean hit rate across conditions may be inflated due to the use of objects as foils in the temporal memory task, making them easier to recognise in later trials) and a correct rejection rate of 83% (Table 2). A 2 × 2 (Object × Direction; see Methods) mixed ANOVA on hit rate failed to reveal any significant main effects or interactions, *F*’s < 2.50, p’s > 0.12, *η_p_*^2^’s < 0.07. Thus, recognition memory did not differ according to whether the object was seen first or second in the room.

Mean accuracy in the context memory task (33%) did not differ from chance, *t*(39) = 0.17, p = 0.87, *d* = 0.03 (Table 3). A 2 × 2 (Object × Direction) mixed ANOVA failed to reveal any significant main effects or interactions, *F*’s < 0.21, p’s > 0.65, *η_p_*^2^’s < 0.01. Therefore, context memory did not differ from chance, and was not modulated by whether the object was seen first or second within the room.

#### 2.2.3 Time and distance between objects at encoding

We next focussed on the mean time and path distance between objects within- vs across-room. If the time (and distance) between objects is shorter within-context relative to across-context then this might explain the pattern of results we observed. Participants may have better memory for objects seen closer together in time and space than those seen further apart, regardless of the presence of any spatial boundaries. Note that the VR environment was designed such that the straight line distance between objects within-room was 10.2 virtual metres (vm) and across-rooms was 8.7 vm. As such, the straight line distance was further within-room than across-rooms. However, it is the path distance and time between objects that may also be critical.

Due to a bug in the VR Study task (resolved in Experiments 2–3), we only had accurate time and path distance data from 10 participants in Experiment 1 (4 from Group 1 and 6 from Group 2). In all 10 participants, the time (mean: 7.75 sec vs 10.72 sec) and path distance (mean: 7.86 vm vs 10.46 vm) was shorter between objects within- than across-rooms. Note, the shorter path distance than straight line distance within-room is due to how the location for each is measured. The straight line distance is measured from the exact location of the object in the environment. The path distance is measured from the location the participant was in when the object question was triggered. These are not identical, as the question was triggered just before the participant reached the edge of the table. Experiment 1 therefore can’t rule out the possibility that the temporal memory effect is driven by this difference in time and path distance (though cannot be explained in relation to straight line distance).

### 2.3 Discussion

Experiment 1 revealed that participants were more accurate when judging which object came next (or before) in a sequence if they were seen in the same room relative to if they were seen in adjoining rooms. This effect is seen despite the temporal judgement always testing sequentially presented objects (i.e., which object came immediately next or before in the sequence). Further, though participants were required to navigate through the rooms, the spatial context was not directly relevant to the encoding task (semantic categorisation) or the temporal memory task. Finally, participants performed at chance in the context memory task, suggesting they could not explicitly remember what room the object was seen in. Thus, it would seem that participants’ memory for object order was modulated by spatial context (and the presence of spatial boundaries) despite no explicit memory for these spatial contexts at retrieval. However, one key issue needs to be addressed, as the time and path distance was shorter between objects within- than across-context.

## 3 Experiment 2

Experiment 2 was designed to replicate Experiment 1, with several important changes. First, the experiment was split into two separate Study-Test blocks, resulting in fewer objects/rooms to encode during each block. This was done in order to potentially improve both temporal and context memory accuracy. Second, the size of each room was doubled (with the number of total rooms halved). This increased the distance between objects within-room relative to across-room, with the intention of removing (or reversing) the time/path distance confound present in Experiment 1. Importantly, each half of these larger rooms had different wallpaper. As such, the background visual information at the time of encoding each object was different for both within-room and across-room object pairs. Finally, the direction of the temporal order question (i.e., “which object came next?” vs “which object came before?”) became a within-subject manipulation. In one of the Study-Test blocks, participants were asked the “next” question and in the other they were asked the “before” question.

### 3.1 Methods

Experiment 2 was identical to Experiment 1 with the following exceptions.

#### 3.1.1 Participants

From Experiment 1, collapsing across Groups 1 and 2, we calculated a Cohen’s *d* of 0.49 for the difference in within-context vs across-context temporal order judgements. To achieve a power of 0.90, this requires 37 participants. 42 participants (21 Male) were recruited in Experiment 2. By self-report 6 participants were left-handed and the remainder right-handed. In total, 6 participants did not finish the experiment, leaving 36 participants with a mean age of 23.4 (SD = 4.6).

#### 3.1.2 Materials

The VR environment was modified by removing the walls between alternate rooms (i.e., rooms 1 and 2 became a single room, as did rooms 3 and 4, etc.), creating half the number of rooms that were double the size. The two tables were situated close to each doorway in order to maximise the distance between objects within- than across-rooms. The wallpaper was kept the same as in Experiment 1, such that each half room had different wallpaper from the other half of the room. This was done in order to minimise the background similarity when encoding each object within- vs across-rooms. The same number of objects was seen at Study, but split between two separate Study-Test phases. The order of objects at Study was changed from Experiment 1 (though still fixed).

#### 3.1.3 Procedure

Participants navigated the 24 rooms, categorising 48 objects in a single Study block. This was followed by a Test block where 40 old objects (from rooms 3–22) and 24 new objects were presented in the same format as Experiment 1. Participants then performed a second Study block. The rooms were identical between blocks, however participants started in a different room and navigated in the opposite direction to the first Study block (i.e., anti-clockwise if the first block was clockwise). 48 new objects were encoded during the second Study block. This was followed by a final Test block where 40 of these objects and 24 new objects were presented. Participants performed both temporal order questions (i.e., “which object came next?” and “which object came before?”) separately, one in each Test block. The order of encoding blocks and the direction of the temporal order question was counterbalanced across participants, resulting in four counterbalancing permutations. This counterbalancing ensured that, across participants, each object acted as cue and target in both the within-context and across-context condition. Thus, despite the fixed encoding order, each object contributed to both the within-context and across-context condition.

#### 3.1.4 Statistical analyses

For temporal memory, we present a 2 × 2 repeated measures ANOVA with the factors Context (within- vs across-context) and Direction (“which object came next” vs “which object came before”). For recognition and context memory we present 2 × 2 repeated measures ANOVAs with factors Object (first vs second) and Direction (“which object came next” vs “which object came before”). We also present a similar ANOVA of the temporal memory data for completeness.

Finally, for temporal memory, we also present a 2 × 2 × 2 mixed ANOVA (Block × Object × Question order), where Block (1st vs 2nd) and Object (1st vs 2nd) are within-subject factors and Question order (which came next, which came before vs which came before, which came next) is a between-subjects factor. This analysis allows us to assess whether temporal memory performance differed across encoding blocks. Note, a three-way interaction in this ANOVA is equivalent to the main effect of Context seen in the main analysis.

### 3.2 Results

#### 3.2.1 Temporal order memory

Mean temporal memory accuracy was above chance at 43% (Table 1), *t*(35) = 4.43, p < 0.001, *d* = 0.74. Accuracy was therefore similar to Experiment 1, despite the number of objects encoded during one Study block being halved. A 2 × 2 repeated measures ANOVA with factors Context and Direction revealed a main effect of Context, *F*(1, 35) = 8.39, p < 0.01, *η_p_*^2^ = 0.19, with higher accuracy in the within-context than across-context condition (Fig.3B). This effect did not interact with Direction, *F*(1, 35) = 0.43, p = 0.52, *η_p_*^2^ = 0.01 (nor was there a main effect of Direction, *F*(1, 35) = 0.22, p = 0.64, *η_p_*^2^ = 0.01). We therefore replicated the results of Experiment 1 – participants were better at judging temporal order within- than across-context.

A 2 × 2 × 2 mixed ANOVA (Block × Object × Question order) failed to reveal a main effect of Block, *F*(1, 34) = 1.82, p = 0.19, *η_p_*^2^ = 0.05, nor did this factor interact with Object or Question order, *F*’s < 0.16, p’s > 0.69, *η_p_*^2^’s < 0.01. The only significant effect was a three-way interaction between Block, Object and Question order, *F*(1, 34) = 8.12, p < 0.01, *η_p_*^2^ = 0.19 (equivalent to the main effect of Context in the main analysis). A 2 × 2 repeated measures ANOVA with factors Object and Direction revealed a significant interaction, *F*(1, 35) = 8.39, p < 0.01, *η_p_*^2^ = 0.19, identical to the main effect of Context reported above (and no main effects *F*’s < 0.44, p’s > 0.51, *η_p_*^2^ < 0.02).

#### 3.2.2 Recognition and context memory

Recognition accuracy was high, with a mean hit rate of 79% and a correction rejection rate of 87% (Table 2). A 2 × 2 (Order × Direction) repeated measures ANOVA on hit rate failed to reveal any significant main effects or interactions, *F*’s < 1.51, p’s > 0.22, *η_p_*^2^ < 0.05. As in Experiment 1, recognition memory did not differ according to whether the object was seen first or second in the room.

Mean accuracy in the context memory task (33%) did not differ from chance, *t*(35) = 0.32, p = 0.75, *d* = 0.05 (Table 3). A 2 × 2 (Order × Direction) repeated measures ANOVA on hit rate failed to reveal any significant main effects or interactions, *F*’s < 2.30, p’s > 0.13, *η_p_*^2^ < 0.07. As in Experiment 1, context memory did not differ from chance, and wasn’t modulated by whether the object was seen first or second in the room.

#### 3.2.3 Time and distance between objects at encoding

In Experiment 2, we collected accurate time and distance information at encoding for all participants. By increasing the size of each room, the straight line distance between objects within-room (17.9 vm) was greater than across-rooms (10.7 vm). Therefore, whereas the difference in straight line distance between within-room and across-room in Experiment 1 was ∼1 vm, here it was ∼7 vm. Importantly, this increase in distance was maintained for the mean path length between objects. The mean path length was significantly greater within-room (17.55 vm) than across-rooms (13.56 vm), *t*(35) = 14.84, p < 0.001, *d* = 2.51. Experiment 2 therefore produced the same pattern of temporal order results despite the straight line and path distance being longer between objects within- than across-rooms.

However, the time taken to navigate between objects was shorter within-room (11.98 sec) than across-rooms (12.95 sec), *t*(35) = 3.50, p < 0.01, *d* = 0.28. As such, the temporal memory effect seen in Experiments 1 and 2 may still be attributable to this difference in time between objects within- vs across-rooms. To control for this difference, we performed a control analysis that removed pairs of objects (per room) based on the time taken to travel between object 1 and 2 (within-room) vs between object 2 and object 1 in the next room (across-room). We rank-ordered pairs according to the difference between within- vs across-room time, and iteratively removed pairs until the mean time within- vs across-rooms across all remaining trials was equal, or the relationship had been reversed (i.e., longer mean time within- than across-rooms). This procedure resulted in a mean removal of 3.56 trials per participant per block per condition (range = 1–14; from a total of 20 trials).

Following this paired trial removal procedure, the time within-room (12.06 sec) was longer than across-rooms (11.62 sec), *t*(35) = 27.75, p < 0.001, *d* = 0.14. Thus, we reversed the time difference seen in the main analyses of Experiment 1 and 2. Despite this, a 2 × 2 (Context × Direction) repeated measures ANOVA on temporal order accuracy (Table 1) revealed a main effect of Context, *F*(1, 35) = 6.72, p < 0.05, *η_p_*^2^ = 0.16, with higher accuracy within-room than across-room (Fig.3D). This effect did not interact with Direction, *F*(1, 35) = 1.48, p = 0.23, *η_p_*^2^ = 0.04 (nor was there a main effect of Direction, *F*(1, 35) = 0.18, p = 0.67, *η_p_*^2^ < 0.01). Therefore, the temporal order difference was seen in Experiment 1 and 2, regardless of whether the time, straight line distance, or path distance between sequential objects was longer or shorter within-room than across-rooms.

### 3.3 Discussion

Experiment 2 replicated Experiment 1. Temporal order accuracy was higher for objects seen in the same room than adjoining rooms. This was seen despite the straight line distance and path distance between successive objects being longer within-room than across-room. Further, the effect remained when we performed an analysis to control for the time between successive objects. The effect was also seen despite the two halves of each room having different wallpaper, such that the background scene when performing the object categorisation task was different within-room and across-room. Thus, objects become more strongly associated by virtue of their shared context, despite being separated in both space and time.

However, Experiments 1–2 may still suffer from one further issue. When a participant interacted with an object, the next object was immediately present on the next table. When interacting with the first object in a room, the second object in the room (within-context) could be seen immediately (or shortly after participants turned to face the next table). When interacting with the second object in a room, the first object in the next room (across-context) would only be seen once the participant moved through the doorway. Thus, although we controlled for the time between interacting with each object within- vs across-room, the time between viewing each object was not controlled.

## 4 Experiment 3

We conducted a final experiment to control for the viewing time of objects within- vs across-room. When a participant interacted with an object, a question mark would appear in the location that the next object would be seen. Participants were required to walk up to the question mark. Once close enough, the same prompt window would appear, as in Experiments 1–2, asking whether the object was man-made or natural. Only at the point when the prompt box appeared would the question mark be replaced by the object to be encoded. Thus, the time the object was seen was identical to the time the encoding task prompt box was triggered.

### 4.1 Methods

Experiment 3 was identical to Experiment 2 with the following exceptions.

#### 4.1.1 Participants

41 participants (13 Male) were recruited. By self-report 2 participants were left-handed and the remainder right-handed. 3 participants did not finish the experiment, leaving 38 participants with a mean age of 22.2 (SD = 4.4).

#### 4.1.2 Procedure

The procedure was identical to Experiment 2, apart from a question mark would appear in the location of the next object to be encoded. Only when the participants triggered the encoding question would the question mark be replaced by the object.

### 4.2 Results

#### 4.2.1 Temporal order memory

Mean temporal memory accuracy was above chance at 48% (Table 1), *t*(37) = 8.07, p < 0.001, *d* = 1.31. A 2 × 2 repeated measures ANOVA with factors Context and Direction revealed a main effect of Context, *F*(1, 37) = 15.73, p < 0.001, *η_p_*^2^ = 0.30, with higher accuracy in the within-context than across-context condition (Fig.3C). This effect did not interact with Direction, *F*(1, 37) = 1.89, p = 0.18, *η_p_*^2^ = 0.05 (nor was there a main effect of Direction, *F*(1, 37) = 0.56, p = 0.46, *η_p_*^2^ = 0.02). We therefore replicated the results of Experiments 1–2 – participants were better at judging temporal order within- than across-context.

As in Experiment 2, a 2 × 2 × 2 mixed ANOVA (Block × Object × Question order) failed to reveal a main effect of Block, *F*(1, 36) = 0.17, p = 0.69, *η_p_*^2^ < 0.01, nor did Block interact significantly with Object or Question order, *F*’s < 0.55, p’s > 0.46, *η_p_*^2^’s < 0.02. The only significant effect was a three-way interaction between Block, Object and Question order, *F*(1, 36) = 15.37, p < 0.001, *η_p_*^2^ = 0.30 (equivalent to the main effect of Context in the main analysis). A 2 × 2 repeated measures ANOVA with factors Object and Direction revealed a significant interaction, *F*(1, 37) = 15.73, p < 0.001, *η_p_*^2^ = 0.30, identical to the main effect of Context reported above (and no main effects *F*’s < 1.90, p’s > 0.17, *η_p_*^2^ < 0.05).

#### 4.2.2 Recognition and context memory

Recognition accuracy was high, with a mean hit rate of 86% and a correction rejection rate of 93% (Table 2). A 2 × 2 (Order × Direction) repeated measures ANOVA on hit rate failed to reveal any significant main effects or interactions, *F*’s < 1.21, p’s > 0.27, *η_p_*^2^ < 0.04. As in Experiments 1–2, recognition memory did not differ according to whether the object was seen first or second in the room.

Mean accuracy in the context memory task (32%) did not differ from chance, *t*(37) = 0.85, p = 0.40, *d* = 0.14 (Table 3). A 2 × 2 (Order × Direction) repeated measures ANOVA on hit rate revealed a main effect of object 1 versus object 2, *F*(1, 37) = 4.97, p < 0.05, *η_p_*^2^ = 0.12. Neither the main effect of Direction, *F*(1, 37) = 0.30, p = 0.86, *η_p_*^2^ < 0.01, nor the interaction between Order and Direction, *F*(1, 37) = 0.43, p = 0.52, *η_p_*^2^ = 0.01, reached significance. Given that performance did not differ from chance in any of the four conditions separately, *t*’s < 1.97, p’s > 0.06, and no effect of Order was seen in Experiments 1 and 2, the main effect of Order seen here is most likely a Type I error. In sum, as in Experiments 1–2, context memory did not differ from chance (though some evidence was seen for variation across object order, unlike in Experiments 1–2).

#### 4.2.3 Time and distance between objects at encoding

As in Experiment 2, the straight line distance between objects within-room (17.9 vm) was greater than across-rooms (10.7 vm). Also in line with Experiment 2, the mean path length was significantly greater within-room (17.51 vm) than across-rooms (13.45 vm), *t*(37) = 27.99, p < 0.001, d = 3.39. Experiment 3 therefore replicated the results of Experiments 1–2 despite straight line and path distance being longer between objects within- than across-rooms.

In line with Experiment 2, the time taken to navigate between objects was shorter within-room (13.33 sec) than across-rooms (15.15 sec), *t*(37) = 7.12, p < 0.001, d = 0.56. We performed the same control analysis as in Experiment 2, selectively removing pairs of trials based on the time taken to travel between objects within-room versus across-room. This procedure resulted in a mean removal of 5.54 trials per participant per block per condition (range = 1–18; from a total of 20 trials).

Following this paired trial removal procedure, the time within-room (13.58 sec) was longer than across-rooms (13.20 sec), *t*(37) = 6.23, p < 0.001, *d* = 0.12. Thus, we reversed the time difference seen in the main analyses of Experiments 1–3. Despite this, a 2 × 2 (Context × Direction) repeated measures ANOVA on temporal memory accuracy (Table 1) revealed a main effect of Context, *F*(1, 37) = 11.52, p < 0.01, *η_p_*^2^ = 0.24, with higher accuracy within-room than across-room (Fig.3E). This effect did not interact with Direction, *F*(1, 37) = 2.05, p = 0.16, *η_p_*^2^ = 0.05 (nor was there a main effect of Direction, *F*(1, 37) = 0.23, p = 0.64, *η_p_*^2^ < 0.01). Therefore, Experiment 3 replicated the results of Experiment 2 for both the main temporal memory analysis, and the analysis to control for time between objects within- versus across-rooms.

### 4.3 Discussion

Experiment 3 replicated Experiments 1–2. This was despite tightly controlling for the time each object was seen. Thus, across three experiments we show better temporal memory for objects seen within the same room relative to those seen in adjacent rooms despite controlling for (1) the straight line, (2) the path distance, (3) the encoding time and (4) the viewing time between objects within- versus across-rooms.

## 5 Computational model

The results of Experiments 1–3 provide clear evidence for the role of spatial boundaries in the formation of event memories. Items encountered within a single spatial context are more readily associated than items encountered in adjacent spatial contexts, even when similarly distanced in both space and time. We next present a simple computational model to account for these results. The model makes as few assumptions as possible with regard to the potential mechanism driving the effect. The intent was to develop the most parsimonious model that is capable of parametrising the spatial boundary effect.

We built upon an existing class of memory models, where items become associated with a ‘context’ signal present at encoding (e.g., Bower, 1972, Burgess and Hitch, 1999, Estes, 1950, Howard and Kahana, 2002, Raaijmakers and Shiffrin, 1981). This context signal varies over time, such that items presented in a sequence will each become associated with a different context. Because the context signal drifts over time, items encountered closer together in time will be associated with more similar contexts than items encountered further away in time. Importantly, we allow the rate of change in this context signal to vary according to external stimuli. Specifically, the rate of change increases in the presence of a spatial boundary, resulting in greater differentiation in contexts between adjacent objects encountered in separate rooms relative to those encountered in the same room. This simple addition to the model captures the temporal memory effect seen in Experiments 1–3.

### 5.1 Methods

**Fig. 4:**
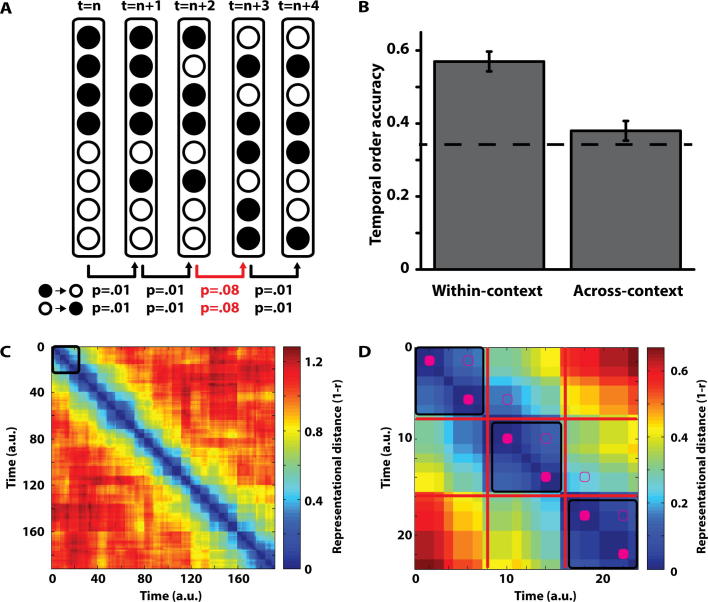
Computational model. (A) Schematic of the model, showing the ‘context vector’ and transition probabilities at 5 arbitrary timepoints. The transition probabilities increase in the presence of a ‘spatial boundary’, as shown between timepoints t = n + 2 and n + 3 (highlighted in red). Note, the example ‘context vectors’ are for illustrative purposes and show a higher rate of change between timepoints than the actual model. (B) Mean accuracy for the 10 model iterations for the within-context and across-context conditions. Error bars represent ±1 standard error of the mean. The dotted line shows chance level of performance (0.33, given three-alternative forced choice). Note, the mean level of performance across conditions is set to 0.45 in each iteration to match the behavioural performance in Experiments 1–3. (C) Representational distance (1 − r) for each timepoint relative to all other timepoints for a single iteration of the model. The black square in the top left shows the area magnified in (D). (D) A magnified view of the representational distance across three ‘rooms’. The 7 timepoints of each square are shown with black squares. Red lines represent a ‘spatial boundary’ where the rate of change in the context vector is increased. The solid magenta squares highlight when each object is ‘presented’ and the non-solid magenta squares highlight the representational distance between each successive object pairing, highlighting the smaller distance within-room than across-room. (For interpretation of the references to colour in this figure legend, the reader is referred to the web version of this article.)

As in Estes (1950), we explicitly modelled a time-varying ‘context’ representation at encoding. The context was a vector of 100 ‘features’ that each moved independently and stochastically between two binary states (0 and 1) over time according to a specified matrix of transition probabilities (i.e., a Markov model). Initially, each feature was randomly assigned to one of the binary states with equal probability. At subsequent timepoints, the ‘baseline’ probability of a feature transitioning from 0-to-1 or 1-to-0 was 0.01. As such the population of feature states – i.e., the context vector – drifted stochastically over time (Fig.4A). The baseline transition probability was chosen to ensure a relatively slow drift across time. Future studies would be needed to accurately estimate the ‘real world’ drift rate.

We simulated 191 timepoints for each iteration of the model (see below). 48 objects were ‘presented’ at fixed intervals across these timepoints. The first object was presented at the 2nd time-point, and each successive object was presented 4 timepoints thereafter. To simplify the model, we presumed the association between each object presented and the context vector at that timepoint was maximal (i.e., there is no variation in encoding strength between the object and its context).

The key manipulation we made to incorporate the effect of spatial boundaries was to allow the rate of change of the context vector to vary across time. Every 8 timepoints (for a single timepoint), we changed the probability of each feature making a transition between states from 0.01 to 0.08. This served to increase the rate of change of the context vector for these timepoints. The increase in transition probabilities was chosen to ensure a clear distinction between contextual representations across rooms. Future studies will be needed to precisely measure this increase, relative to the baseline transition probabilities. We defined these timepoints as ‘spatial boundaries’, with the 7 timepoints between each spatial boundary corresponding to a single ‘room’. Each object was presented on the 2nd and 6th timepoint in each room, ensuring every object was separated by three timepoints irrespective of the within-context vs across-context manipulation (Fig.4D). Thus, as in Experiments 1–3, the model was presented with two objects, then a spatial boundary, continuously for 24 ‘rooms’.

At retrieval, we compared the dissimilarity (referred to as ‘representational distance’) between the context vector associated with the cue object at encoding relative to the three choice alternatives presented (i.e., the correct adjacent object and the two foils). The two foils were randomly chosen with the same constraints as Experiments 1–3. We calculated Pearson’s r for the cue context relative to the three choice object contexts, defining representational distance as 1 − r (Fig.4C and D). For each trial, we calculated this distance measure and calculated the proportion of the distance for the correct choice relative to the foil with the next shortest distance. We then set a threshold based on these proportions across all trials (irrespective of within- vs across-context condition) so that the model was correct in ∼45% of trials (i.e., approximately equating performance to Experiments 1–3). For simplicity, we do not model a formal ‘decision making’ process at retrieval (e.g., Ratcliff, 1978). Though such a mechanism could readily be incorporated into the model, we wanted to focus explicitly on how a time-varying context representation could account for our findings. We ran 10 differently seeded iterations of the model and calculated the accuracy for within-context and across-context accuracy for each iteration.

The MATLAB code to run this model, and produce the figures shown in Fig. 4, is freely available on Figshare (http://dx.doi.org/10.6084/m9.figshare.1609804.v3).

### 5.2 Results

How can such a time-varying context signal account for our results? Presuming the context signal drifts at a uniform rate across time (p = 0.01), no differences in accuracy would be expected between the within-context and across-context conditions. This is because the similarity in contexts between the cue and correct answer will be similar between the two conditions. Indeed, when we did not modulate the rate of change in the presence of an event boundary, performance in the within-context (47%) condition was comparable to the across-context condition (48%).

In our model, we varied the rate of contextual drift in the presence of a spatial boundary (increasing from p = 0.01 to p = 0.08). In other words, the context signal changes more quickly when you walk through a doorway than when walking across a room. Allowing the rate of contextual drift to vary in the presence of a spatial boundary decreases the similarity in contexts between two adjacent objects across-rooms relative to within-room. As object representations are directly associated with this context signal, and accuracy is driven by the similarity of the context signal at encoding, this results in more accurate temporal memory within-context (57%) than across-context (38%; Fig.4B). Our model therefore appropriately captures the main behavioural finding in Experiments 1–3.

## 6 General discussion

Our continuous sensory experience is thought to be segmented into discrete events that are subsequently encoded in long-term episodic memory. Across three experiments we show that spatial boundaries play an important role in this segmentation process. Specifically, the presence of a spatial boundary affects long-term memory for the temporal order of objects. When two objects are sequentially presented in the same room, temporal memory is more accurate than when the two objects are presented in adjoining rooms. In other words, long-term temporal memory is disrupted when two objects are separated by a spatial boundary. Further, we provide a parsimonious algorithmic model to explain this effect, allowing for the parameterisation of the effect in future studies.

Previous research has shown that moving through a spatial boundary (doorway) disrupts short-term memory for objects from the previous room, the ‘location updating effect’ (Radvansky & Copeland, 2006). In these experiments, memory for the object they are currently ‘carrying’ (i.e., the second object in the room) is disrupted when probed shortly after moving through a doorway relative to the same object when probed midway through the room. Could the simple act of forgetting the last object in the room account for our long-term temporal memory effect? If the associative strength between two objects is modulated by their availability in working memory, it is possible this would lead to stronger associations between objects seen in the same room relative to those seen in adjoining rooms. This is because the last object in the previous room will no longer be available in working memory when the first object in the next room is encountered.

The most obvious predicted long-term consequence of the short-term ‘location updating effect’ would be that, if the last object seen in each room was removed from working memory more rapidly than the first object in each room, it should have less opportunity to be stored in long term memory so that recognition memory for these items would be impaired compared to those seen first in the room. Collapsing across all three experiments, we could find no difference in recognition hit rate between objects seen first or second in each room, *t*(113) = 0.55, p = 0.59, d = 0.03. Thus, we could find no evidence for a simple consequence of the ‘location updating effect’ on long-term memory, at least in relation to recognition memory for single items. Rather, while the transition of information from working memory to long-term memory may be triggered by crossing a spatial boundary, the efficiency of the transition is not affected (consistent with Barreau & Morton, 1999).

This finding seems potentially at odds with the ‘location updating effect’. If removal of items from working memory by crossing a boundary shortly after encoding does not affect their encoding into long-term memory, why is short-term memory performance affected? Why can’t the newly encoded long-term representation support performance in the absence of a working memory representation? The most obvious resolution is that performance based on long term memory representations is worse than that based on working memory, perhaps due to the large number of similar items already encoded into long-term memory, or because of impaired retrieval from long-term memory when in a new room (e.g., context-dependent memory; Gooden and Baddeley, 1975, Thomson and Tulving, 1970, Tulving and Thompson, 1973, though see Radvansky et al., 2011).

Although the present spatial boundary effect is unlikely to be driven up the ‘location updating effect’ *per se* (i.e., recent objects being removed from working memory), both effects are likely to be driven by the same underlying event segmentation process. Whereas event segmentation may remove specific items from working memory (affecting short-term memory), the same process must also modulate the associative strength between items (either directly, or indirectly by a shared contextual representation, see Computational Model and further discussion below), thus affecting long-term temporal memory. These two apparently independent effects of event segmentation, giving rise respectively to distinct short-term and long-term effects, may be mutually consistent within the context of a more sophisticated model of the interaction between working memory and long term memory. Thus, if we assume that associations between items and contextual representations (which may include item information) are formed by both being present simultaneously in working memory, then the location updating effect on working memory means that within-room items will be associated to similar contextual representations (while co-active in working memory), whereas items in contiguous rooms will be associated to distinct contextual representations. This possibility corresponds to the idea that working memory comprises the active part of long term memory (e.g., Cowan, 1995, Fuster, 1997, Melton, 1963) and that plasticity is Hebbian in associating co-active elements (Hebb, 1949). It also corresponds to the idea that processing in working memory contributes directly to the formation of episodic memories, consistent with the idea that working memory contains an ‘episodic buffer’ (Baddeley, 2000).

Such long-term event segmentation effects have been shown in relation to non-spatial event boundaries (DuBrow & Davachi, 2013). However, the role of spatial boundaries in relation to the structure of long-term episodic memory has not been explored. In the case of DuBrow and Davachi (2013), event boundaries were defined by a change in the stimulus-type (faces vs objects) and in the categorisation task performed to each stimulus-type (see Davachi & DuBrow, 2015 for a review). Thus, the event boundaries were explicitly linked to the encoding task. Here, the event boundaries were incidental to the object encoding task. Although participants had to navigate through the rooms, the encoding task related to each object remained constant throughout. Thus, we show that changes in the background context (as opposed to the encoding task) also affects memory for object sequences.

One important difference between the current experiments and those of DuBrow & Davachi is the nature of the temporal memory task. Whereas we asked “which object came next (or before)?”, they presented two objects and asked “which object was more recent?”. Although seemingly subtle, it could be that these tasks are solved with different mechanisms. For example, whereas DuBrow & Davachi’s recency judgement could utilise an item-based familiarity signal (if one presumes that such a signal decreases over time, such that objects seen further back in time will generate a smaller familiarity signal), our sequential judgement could be inferred from direct associations between objects, or between objects and an associated ‘context’ signal (as we have explicitly modelled). In other words, it is possible that our “temporal memory” task could be solved without any specific “temporal” memory representation. However, here we were not interested in ‘temporal memory’ *per se*, but the effects of spatial boundaries on episodic memory; our ‘temporal memory’ task being the most effective means to assess such an effect.

Given the key role spatial context is thought to play in episodic memory (Burgess et al., 2002, O’Keefe and Nadel, 1978, Tulving, 1983), it is perhaps surprising that the relationship between spatial boundaries and the structure of episodic memory has not been previously assessed. The emphasis of previous work has often related to the information encoded in an event engram within the hippocampus – i.e., whether ‘event’ representations are inherently spatial in nature (e.g., Burgess et al., 2001, Byrne et al., 2007, Cohen and Eichenbaum, 1993, Robin et al., 2016, Ryan et al., 2000). Here, we focus on the role of spatial boundaries in the formation of event representations. When two objects are separated in space and time, what environmental factors increase/decrease the likelihood that they will be associated, and therefore encoded in the same event representation? The formation of associations between information separated in space and time is thought to be a key function of the hippocampus (Staresina and Davachi, 2009, Wallenstein et al., 1998). Here we show that spatial boundaries play a role in determining the extent to which disparately presented objects become associated.

Models of memory often include a time-varying ‘context’ representation (e.g., Bower, 1972, Estes, 1950, Raaijmakers and Shiffrin, 1981). When a stimulus is encountered, it is associated with the context representation at that specific time-point. These models have been used to explain a wide body of behavioural phenomena, from spontaneous recovery in traditional associative learning (Estes, 1955), to primacy and recency in short-term memory (Burgess & Hitch, 1999), to forward recall in free recall tasks (Howard & Kahana, 2002). This class of model is also readily able to support temporal order memory judgements (Bower, 1972). We infer that two objects were seen closer together in time as they are each associated with context representations that are more similar than two objects seen further apart in time. This type of model, in which the context signal drifts relatively separately from the external surroundings, is consistent with the observation of slow ‘drift’ in the population firing of pyramidal cells in CA1 of the rodent hippocampus across time, when controlling for the location and behaviour of the rodent (Mankin et al., 2015, Manns et al., 2007, Rubin et al., 2015).

In the present experiments participants had better memory for object pairs seen within the same room relative to adjoining rooms, despite controlling for both the time and distance between object pairs. Thus, a model with uniform contextual drift cannot account for this effect. As such, we allowed the rate of contextual drift to vary in relation to the presence of a spatial boundary. This allowed the context representation to change more rapidly in the presence of a spatial boundary relative to the baseline level of drift. This simple addition was able to fully account for the key finding in Experiments 1–3 (Fig. 4).

What might drive this change in context signal across rooms? The most likely possibility is the external environmental features themselves. For example, the layout of the room, the colour of the wallpaper, the locations of the doors, etc. might all contribute to a general ‘context’ representation in the hippocampus and surrounding medial temporal lobe. This idea is similar to the way in which the Temporal Context Model allows the context signal at timepoint *t* + 1 to incorporate item information from timepoint *t* (Howard and Kahana, 2002, Polyn and Kahana, 2008). However, here the effect would need to be driven by the surrounding spatial context, as opposed to the items themselves, as we controlled for the time between objects within- vs across-rooms (in the control analyses of Experiments 2–3). The neural signature of this might be the phenomenon of “remapping”, whereby place cells in the rodent hippocampus change their firing dependent on the context (Anderson and Jeffery, 2003, Bostock et al., 1991, Leutgeb et al., 2005, Wills et al., 2005).

Importantly, the extent of remapping is driven by environmental differences such as the locations of boundaries, the colour of the walls or the smell of the environment. Note, in Experiment 2 the two halves of each room had different wallpaper and the two tables in each room were never the same. Thus, at the time of performing the object encoding task, the background scene was dissimilar both within- and across-room. If a context signal was changing in relation to the surrounding context, it would appear that basic visual features (e.g., the colour of the walls) do not contribute (or contribute minimally). Thus the present experiments indicate a specific role for the walls in separating the different rooms into different contexts. This may reflect the specific role played by physical boundaries in spatial navigation – such that enhanced encoding of doorways by hippocampal place cells is seen (Spiers et al., 2013), supporting subsequent navigation (Stachenfeld et al., 2014), while their spatial receptive fields within a given context are specifically determined by the proximal boundaries (Hartley, Burgess, Lever, Cacucci, & O’Keefe, 2000).

Note, our model makes no predictions in relation to whether the context signal passively “drifts” at variable rates determined by the external environment, or whether it changes as a direct response to perceptual changes in the external environment. It is an algorithmic model that enables the quantification of the spatial boundary effect. Finally, it is interesting to note that participants were at chance in the context judgement task. Although they could remember the order that objects were presented, they could not remember what room the object was seen in. Thus, if participants were solving the temporal memory judgement via an associated context signal (as we have explicitly modelled), they would appear to not have conscious access to such a signal (at least in the present experiments).

An alternative explanation for the doorways effect is that the act of walking through a spatial boundary results in the encoding of information encountered pre-boundary into long-term memory. For instance, the objects (as well as surrounding contextual information) might be actively maintained in working memory, akin to the idea of an “episodic buffer” (Baddeley, 2000), and subsequently encoded into long-term memory in the presence of a spatial boundary (see also Barreau & Morton, 1999). Interestingly, recent fMRI research has shown an increase in hippocampal BOLD response at the offset of video clips is predictive of subsequent memory for the videos (Ben-Yakov and Dudai, 2011, Ben-Yakov et al., 2013, Ben-Yakov et al., 2014). Extending this research to the current experiments, the prediction would be that an increase in hippocampal BOLD should be seen when participants walk through a doorway (equivalent to the end of a video clip in the studies by Ben-Yakov and colleagues). The behavioural doorways effect should correlate with this increase, as opposed to hippocampal activity at the time of encoding the individual objects. It is worth noting that Ezzyat and Davachi (2011) did not see such a relationship in their fMRI study of narrative structures. However, the spatial boundaries in the present experiment might be more salient event boundaries than the perhaps more subtle narrative manipulation used by Ezzyat & Davachi (i.e., “a while later” vs “a moment later”). As such, hippocampal activity changes might be more prominent, and more predictive of subsequent temporal memory, when using the doorway manipulation relative to narrative manipulations.

Despite the evidence presented that spatial boundaries play a key role in shaping long-term episodic memory, it is important to note that space is unlikely to be the sole determining factor. The work of Davachi and colleagues suggests a role for short vs long passages of time in narratives, where no clear spatial context manipulation is present (Ezzyat & Davachi, 2011). Further, switches in task and/or stimulus-type appear to also segregate our experience and have lasting consequences on temporal order memory (DuBrow and Davachi, 2013, DuBrow and Davachi, 2014). Finally, ‘event’ representations can also be constructed through shared content, where no obvious overlap in spatiotemporal context is present. For example, independently encoded but overlapping pairwise associations can be integrated into coherent ‘event’ representations when all possible pairs are explicitly encoded (Horner et al., 2015, Horner and Burgess, 2014). Thus, hippocampus dependent ‘event’ representations (Burgess et al., 2001, Cohen and Eichenbaum, 1993, Eichenbaum et al., 2007, O’Keefe and Nadel, 1978, Ryan et al., 2000) can be formed via shared context or content, allowing for subsequent recollection (Jacoby et al., 1993, Tulving, 1983, Yonelinas, 1994).

Regardless of the precise mechanism, the present experiments provide evidence that the presence of a spatial boundary modulates temporal memory for object sequences. Participants are better at remembering “which object came next (or before)” when the two objects were encountered in the same room relative to when they were encountered in adjacent rooms. This effect was seen despite controlling for both the time and path distance between object pairs within- versus across-room. We therefore provide the first evidence that the structure of long-term episodic memory is shaped by participants’ surrounding spatial context.

## Figures and Tables

**Table 1:** Temporal memory. Mean (and standard deviation) across Experiments 1–3 and the control analyses of Experiments 2–3 (Experiments 2-C & 3-C) for the Within-context and Across-context conditions across the two temporal memory questions “which object came next?” and “which object came before?”.

	Which object came next?		Which object came before?	
Within-context	Across-context	Within-context	Across-context
Experiment 1	0.52 (0.15)	0.42 (0.10)	0.42 (0.15)	0.38 (0.13)
Experiment 2	0.45 (0.17)	0.38 (0.18)	0.46 (0.21)	0.41 (0.16)
Experiment 2-C	0.45 (0.18)	0.38 (0.19)	0.44 (0.21)	0.42 (0.17)
Experiment 3	0.52 (0.18)	0.44 (0.14)	0.49 (0.18)	0.44 (0.15)
Experiment 3-C	0.52 (0.20)	0.41 (0.15)	0.47 (0.19)	0.43 (0.17)

**Table 2:** Recognition memory. Mean (and standard deviation) across Experiments 1–3 for the 1st and 2nd object hit rate and correct rejections (CRs) across the two temporal order questions “which object came next?” and “which object came before?”.

	Which object came next?			Which object came before?		
1st Object	2nd Object	CRs	1st Object	2nd Object	CRs
Experiment 1	0.79 (0.16)	0.76 (0.18)	0.85 (0.15)	0.75 (0.15)	0.73 (0.17)	0.81 (0.19)
Experiment 2	0.79 (0.17)	0.80 (0.14)	0.87 (0.13)	0.78 (0.17)	0.77 (0.15)	0.86 (0.17)
Experiment 3	0.85 (0.19)	0.86 (0.17)	0.93 (0.11)	0.87 (0.16)	0.87 (0.16)	0.93 (0.07)

**Table 3:** Context memory. Mean (and standard deviation) across Experiments 1–3 for the 1st and 2nd objects across the two temporal order questions “which object came next?” and “which object came before?”.

	Which object came next?		Which object came before?	
1st Object	2nd Object	1st Object	2nd Object
Experiment 1	0.32 (0.08)	0.33 (0.08)	0.33 (0.08)	0.33 (0.08)
Experiment 2	0.31 (0.10)	0.33 (0.10)	0.33 (0.07)	0.36 (0.10)
Experiment 3	0.35 (0.10)	0.30 (0.09)	0.34 (0.10)	0.31 (0.10)
